# Structure of the metastatic factor P-Rex1 reveals a two-layered autoinhibitory mechanism

**DOI:** 10.1038/s41594-022-00804-9

**Published:** 2022-07-21

**Authors:** Yong-Gang Chang, Christopher J. Lupton, Charles Bayly-Jones, Alastair C. Keen, Laura D’Andrea, Christina M. Lucato, Joel R. Steele, Hari Venugopal, Ralf B. Schittenhelm, James C. Whisstock, Michelle L. Halls, Andrew M. Ellisdon

**Affiliations:** 1 Biomedicine Discovery Institute, Monash University, Clayton, Victoria Australia; 2 Drug Discovery Biology Theme, Monash Institute of Pharmaceutical Sciences, Monash University, Parkville, Victoria Australia; 3 Monash Proteomics and Metabolomics Facility, Monash University, Clayton, Victoria Australia; 4 Ramaciotti Centre for Cryo-Electron Microscopy, Monash University, Clayton, Victoria Australia; 5 EMBL Australia, Monash University, Melbourne, Victoria Australia; 6 ACRF Department of Cancer Biology and Therapeutics, John Curtin School of Medical Research, Australian National University, Canberra, Australian Capital Territory Australia

**Keywords:** Cryoelectron microscopy, X-ray crystallography

## Abstract

P-Rex (PI(3,4,5)P_3_-dependent Rac exchanger) guanine nucleotide exchange factors potently activate Rho GTPases. P-Rex guanine nucleotide exchange factors are autoinhibited, synergistically activated by Gβγ and PI(3,4,5)P_3_ binding and dysregulated in cancer. Here, we use X-ray crystallography, cryogenic electron microscopy and crosslinking mass spectrometry to determine the structural basis of human P-Rex1 autoinhibition. P-Rex1 has a bipartite structure of N- and C-terminal modules connected by a C-terminal four-helix bundle that binds the N-terminal Pleckstrin homology (PH) domain. In the N-terminal module, the Dbl homology (DH) domain catalytic surface is occluded by the compact arrangement of the DH-PH-DEP1 domains. Structural analysis reveals a remarkable conformational transition to release autoinhibition, requiring a 126° opening of the DH domain hinge helix. The off-axis position of Gβγ and PI(3,4,5)P_3_ binding sites further suggests a counter-rotation of the P-Rex1 halves by 90° facilitates PH domain uncoupling from the four-helix bundle, releasing the autoinhibited DH domain to drive Rho GTPase signaling.

## Abstract (web summary)

Cryo-EM, X-ray crystallography and crosslinking mass spectrometry are harnessed to solve the structure of the full-length Rho-GEF P-Rex1, uncovering a two-layered mechanism of autoinhibition released upon Gβγ and PI(3,4,5)P3 binding.

## Main

Rho GTPases are small GTP-binding proteins (G proteins) of the rat sarcoma (Ras) superfamily that regulate cytoskeletal dynamics, cell motility and the cell cycle^1^. Guanine nucleotide exchange factors (GEFs) promote GTPase cycling from inactive GDP-bound to active GTP-bound forms^2^. GEFs are powerful on-switches for GTPase signaling. As such, GEFs are subject to tight regulation to prevent GTPase hyperactivation and runaway cell growth and motility^2^.

P-Rex1 and its close homolog P-Rex2 are highly conserved GEFs that activate Rho GTPases (Rac1, Cdc42, RhoG) to coordinate cytoskeletal organization and cell motility^3–6^. P-Rex1 overexpression is linked to multiple cancers, including breast cancer and melanoma^7^. P-Rex2 is one of the most commonly mutated proteins in melanoma^8^, pancreatic^9^ and metastatic cancer^10^ and forms a coinhibitory complex with the tumor suppressor PTEN^11,12^.

**Extended Data Fig. 1:**
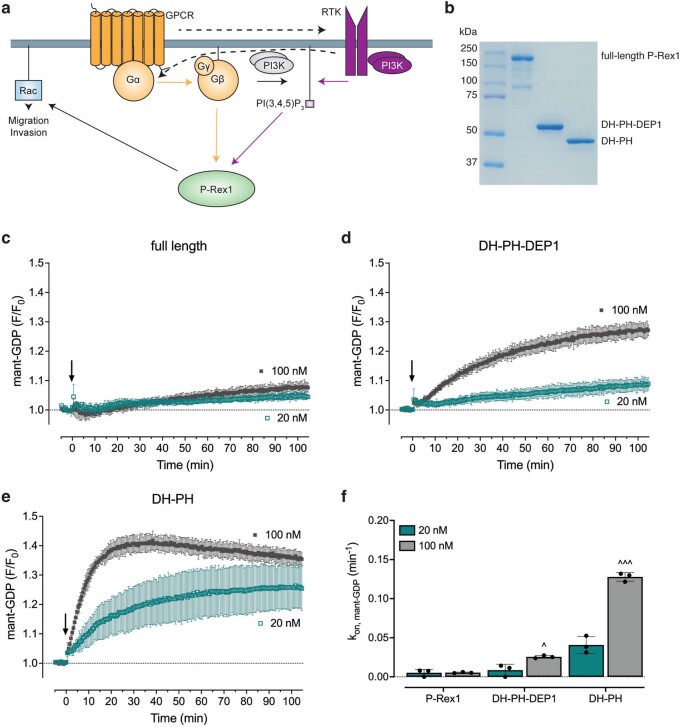
P-Rex1 signalling, variant purification, and activity analysis. **a**. Schematic of P-Rex1 activation. P-Rex1 co-ordinates signalling from GPCRs and RTKs. The key GPCR and RTK effectors, Gβγ and PI(3,4,5)P_3_, respectively, bind to and activate P-Rex1 (solid arrows). Bidirectional cross-talk between GPCRs and RTKs may provide additional pathways to P-Rex1 activation (dotted arrows). For example, Gβγ subunits can generate PI(3,4,5)P_3_ via activation of PI3K; GPCRs can transactivate RTKs to increase PI(3,4,5)P_3_; and RTKs can activate G proteins to release Gβγ subunits^47,48^. **b**. Coomassie-stained SDS-PAGE analysis of purified full-length P-Rex1, P-Rex1 DH-PH-DEP1 (residues 1-502), and P-Rex1 DH-PH (residues 1-404). **c-e**. P-Rex1 GEF activity increases upon truncation of the C-terminal domains. Activity of indicated P-Rex1 variants monitored at 20 nM and 100 nM using mant-GDP activity assay. Symbols show mean and error bars show S.D. (n = 3). **f**. Rate constant (k_obs_) of mant-GDP activity determined from (c-e). Symbols show k_obs_ from independent experiments, bars show mean and error bars show S.D. ^ p = 0.0165 and ^^^ p < 0.0001 versus 20 nM, two-way ANOVA with Šídák’s multiple comparisons test. Numerical data for graphs in c-f are available as source data. Source data

P-Rex family members are autoinhibited under basal conditions and are thought to be synergistically activated at the plasma membrane by receptor tyrosine kinases (RTKs) and G protein-coupled receptors (GPCRs) (Extended Data Fig. 1a)^3^. However, the molecular basis for P-Rex activation by the second messenger signaling molecules PI(3,4,5)P_3_ and Gβγ remains to be determined.

**Fig. 1:**
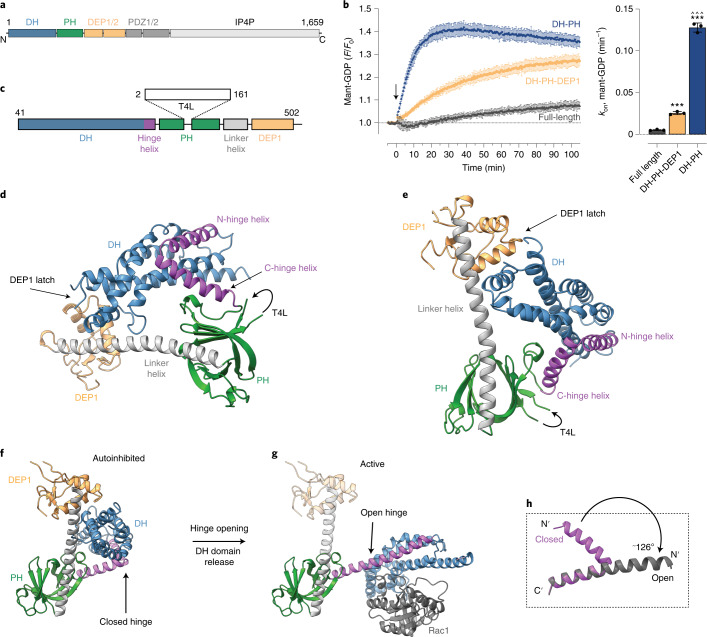
Crystal structure of autoinhibited P-Rex1 DH-PH-DEP1. **a**, P-Rex1 domain layout. **b**, P-Rex1 GEF activity increases upon truncation of the C-terminal domains. Activity of indicated P-Rex1 variants monitored at 100 nM using mant-GDP activity assay. For timecourse graphs, symbols show mean and error bars show s.d. of *n* = 3 independent experiments conducted in duplicate. For bar graphs, symbols show rate constant (*k*_obs_) from independent experiments, bars show mean and error bars show s.d. (*n* = 3). ***P < 0.001 versus full-length (*P* = 0.0082 for DH-PH-DEP1 and *P* < 0.0001 for DH-PH); ^^^ *P* = 0.0005 versus DH-PH-DEP1; repeated measures one-way ANOVA with Tukeyʼs multiple comparisons test. Numerical data for graphs in **b** are available as source data. **c**, Domain layout of the ^ΔN40^DH-PH-DEP1^T4L^ structure highlighting the placement of T4L in the β_3_-β_4_ PH domain loop. **d**, Crystal structure of the P-Rex1 DH-PH-DEP1 (residues 41–305–T4L–323–502) domains in a closed conformation highlighting the DH domain hinge helix and DEP1 latch regions. The placement of T4L is indicated. However, the domain was too flexible to be accurately placed or built into the electron density maps. Instead, comparison of ion-exchange profiles indicates that T4L permitted the purification of a homogenous protein preparation (Extended Data Fig. 2a–d). **e**, Rotated view of **d**. **f**,**g**, Comparison of the autoinhibited P-Rex1 DH-PH-DEP1 structure (**f**) with the active P-Rex1 DH-PH:Rac1 (ref. ^13^) complex (PDB 4YON) (**g**). Upon transition from the autoinhibited to active states, the DH domain hinge helix opens by around 126° flipping the DH domain away from the PH domain to expose the Rac1 binding site. For clarity, the DEP1 domain (light gray and yellow) is modeled onto the active P-Rex1 DH-PH:Rac1 (ref. ^13^) structure (PDB 4YON) to illustrate its position. **h**, Comparison of the DH hinge helix in the open and closed conformations. Source data

P-Rex1 is a 186 kDa multidomain-containing protein with an N-terminal catalytic DH domain, a PI(3,4,5)P_3_-binding PH domain, tandem Dishevelled, Egl-10 and Pleckstrin domain (DEP) domains, tandem PDZ domains and a phosphoinositide-4-phosphatase domain (IP4P) (Fig. 1a)^3^. Several of the P-Rex1 domains have been solved in isolation, or in complex with downstream effectors. For example, P-Rex1 DH-PH domain structures have uncovered a conserved DH domain-mediated mechanism of GDP displacement and the molecular details of the PH domain PI(3,4,5)P_3_ binding pocket^13,14^. More recently, the cryogenic electron microscopy (cryo-EM) structure of the P-Rex1 DEP2-PDZ1/2-IP4P domains in complex with Gβγ determined an extensive binding interface mediated by the PDZ and IP4P domains^15^. Nevertheless, despite this progress, both the P-Rex1 autoinhibitory mechanism and the structural basis of synergistic RTK and GPCR activation have remained elusive.

## Results and discussion

### Structural basis of P-Rex1 autoinhibition

To ascertain the P-Rex1 domains essential for autoinhibition, we compared the activity of full-length P-Rex1 with that of two variants truncated after the DEP1 or PH domains (Fig. 1b and Extended Data Fig. 1b–f). We found that full-length P-Rex1 was essentially inactive, confirming that P-Rex1 is locked in an autoinhibited conformation under basal conditions (Fig. 1b). We triggered small gains in GEF activity by truncating P-Rex1 after the DEP1 domain (Fig. 1b). However, we could drastically increase P-Rex1 activity by further removal of the DEP1 domain (Fig. 1b). We interpreted these data as revealing a two-layered autoinhibition mechanism. The DEP1 domain coordinates the first inhibitory layer, and a second layer is formed by the C-terminal domains locking the DH-PH-DEP1 domains in an autoinhibited conformation.

**Extended Data Fig. 2:**
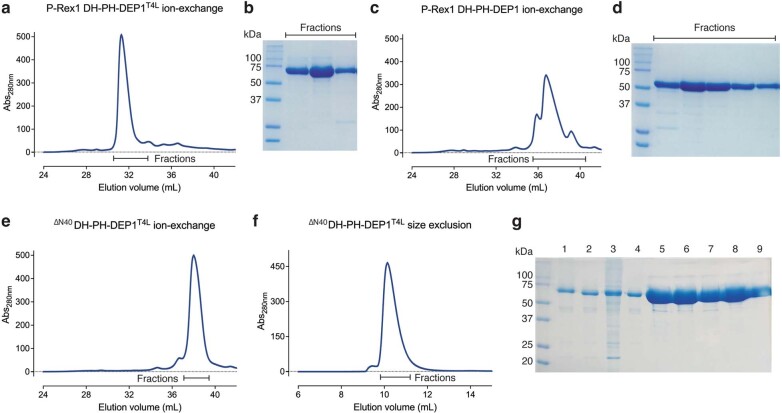
Purification of P-Rex1 DH-PH-DEP1^T4L^. **a**. MonoS ion-exchange chromatography profile of purified P-Rex1 DH-PH-DEP1^T4L^. **b**. Coomassie-stained SDS-PAGE analysis of fractions indicated in (a). Representative SDS-PAGE from two independent purifications. **c**. Comparative MonoS ion-exchange chromatography profile of purified P-Rex1 DH-PH-DEP1. **d**. Coomassie-stained SDS-PAGE analysis of fractions indicated in (c). Representative SDS-PAGE from three independent purifications. Profiles suggest that T4L insertion promoted the purification of a more homogenous protein preparation. **e**. Ion-exchange and **f**. size exclusion chromatography profiles of the ^ΔN40^P-Rex1 DH-PH-DEP1^T4L^ protein utilised to obtain the final 3.2 Å crystal structure. **g**. Coomassie-stained SDS-PAGE analysis of ^ΔN40^DH-PH-DEP1^T4L^ purification (1) nickel elution, (2) overnight (o/n) TEV digest, (3) pellet after o/n TEV and phosphatase treatment, (4) supernatant after o/n TEV and phosphatase digest, (5-6) main-peak MonoS fractions, (7-9) main-peak size exclusion chromatography fractions. Representative SDS-PAGE from three independent purifications.

**Extended Data Fig. 3:**
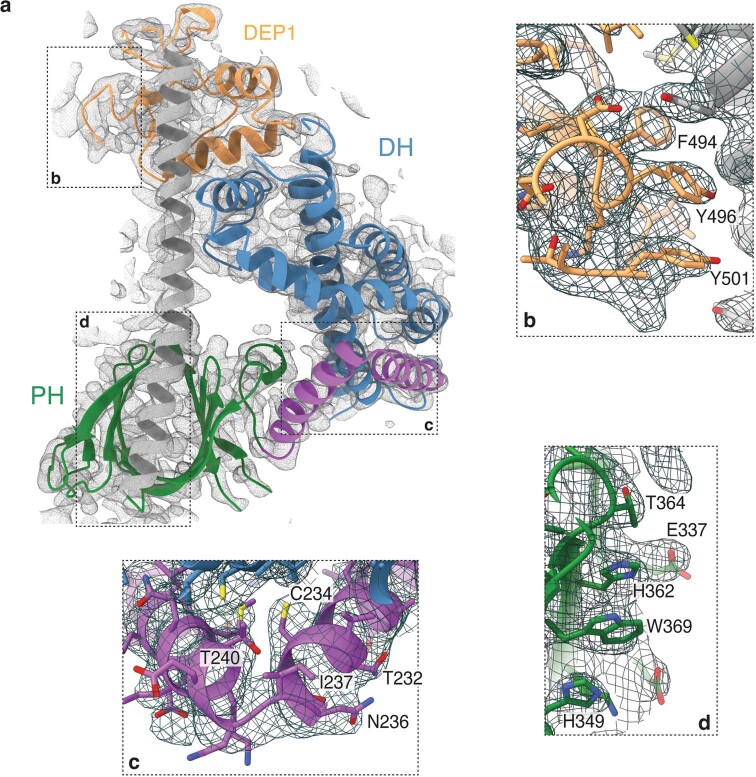
Electron density maps of P-Rex1 ^Δ^^N40^DH-PH-DEP1^T4L^. **a-d**. 2Fo-Fc maps of the P-Rex1 DH-PH-DEP1 structure contoured between 1-1.5σ. Inset regions highlighting the density in the **b**. DEP1 domain, **c**. the DH hinge helix, and **d**. the PH domain. Electron density for T4L was diffuse, preventing the confident placement and refinement of a T4L model. As such, no contacts between T4L and P-Rex1 (either within the P-Rex1 chain or across the crystal lattice) were observed that could affect the conformation of the crystal structure. The T4L domain appears to be positioned within a solvent cavity in the crystal, enabling a high degree of mobility. Regardless, T4L was essential for crystal formation.

To determine the molecular basis for P-Rex1 autoinhibition, we conducted crystallography trials of more than 30 P-Rex1 or P-Rex2 DH-PH-DEP1 constructs incorporating combinations of N-terminal truncations, loop deletions and domain boundary modifications that all failed to produce crystals. Nevertheless, in a breakthrough, splicing T4 lysozyme (T4L) into the unstructured β_3_–β_4_ loop of the PH domain and deleting the first N-terminal 40 residues resulted in diffracting crystals and a final 3.2 Å P-Rex1 DH-PH-DEP1 structure (Fig. 1c–e, Table 1 and Extended Data Figs. 2 and 3). In the crystal structure, the DH-PH-DEP1 domains form a closed triangular topology with the DH domain intercalated into a groove formed by the PH and DEP1 domains and an extended PH-DEP1 linker helix (Fig. 1d,e). This closed conformation positions the PH domain against the DH domain to sterically inhibit the catalytic Rac1 binding surface (compare Fig. 1f with 1g).

**Table 1:** Data collection and refinement statistics

	P-Rex1 ^ΔN40^DH-PH-DEP1^T4L^ (PDB 7RX9)
**Data collection**	
Space group	*P*4_1_22
Cell dimensions	
*a*, *b*, *c* (Å)	151.3, 151.3, 94.3
*α*, *β*, *γ* (°)	90, 90, 90
Resolution (Å)	47.83–3.22 (3.48–3.22)^a^
*R*_merge_	0.117 (1.859)
*R*_pim_	0.053 (0.860)
*I* / σ*I*	7.8 (1.0)
*CC*_1/2_	0.998 (0.358)
Completeness (%)	99.1 (97.6)
Redundancy	6.4 (6.3)
**Refinement**	
Resolution (Å)	47.83–3.22
No. reflections	17,958
*R*_work_ / *R*_free_	0.236/0.262
No. atoms	3,555
Protein	3,525
Ligand/ion	30
Water	0
*B* factors	127.08
Protein	126.76
Ligand/ion	164.78
Water	0
Root mean squared deviations	
Bond lengths (Å)	0.003
Bond angles (°)	0.54

^a^A single crystal was used for the structure (values in parentheses are for highest-resolution shell).

**Extended Data Fig. 4:**
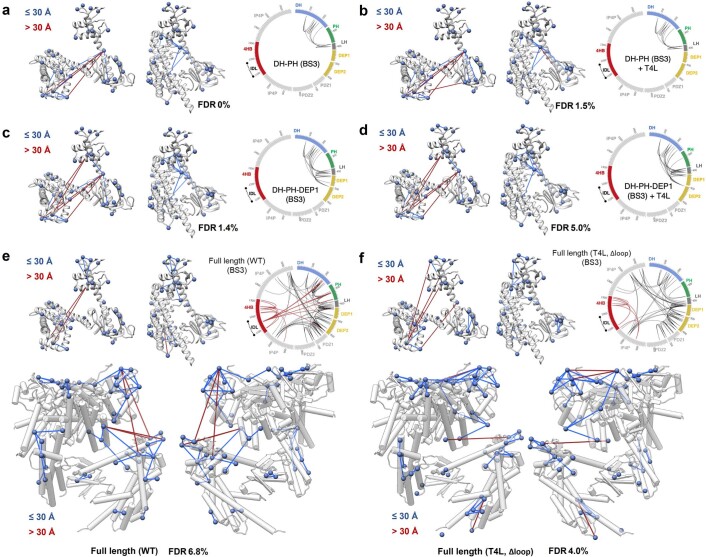
Cross-linking mass spectrometry analysis of P-Rex1. BS^3^ cross-linking constraints and Circos plots of cross-linking mass spectrometry are shown for **a**. the isolated DH-PH domains, **b**. the DH-PH^T4L^ domains, **c**. the DH-PH-DEP1 domains, **d**. the DH-PH-DEP1^T4L^ domains, **e**. full-length wild-type (WT) P-Rex1, and **f**. the full-length ^ΔN40^P-Rex1^T4L, Δloop^ construct utilised for cryo-EM studies. Loop refers to residues 1119-1211. Lysine Cβ atoms are shown as blue spheres with blues lines indicating a compatible constraint (<30 Å between lysine Cβ atoms) and red lines indicating an incompatible constraint (>30 Å between lysine Cβ atoms). Constraints are mapped onto active (left) or autoinhibited (right) models of the DH-PH-DEP1 domains. Cross-links are in excellent agreement with the closed conformation observed in our DH-PH-DEP1 crystal structure. These data indicate that the closed conformation is stably populated in solution in the presence or absence of T4L. Conversely, cross-links frequently exceeded the allowable constraint distance when modelled on the active conformation. Full-length P-Rex1 displays a similar cross-linking pattern in the absence or presence of T4L (e-f). Interestingly, incompatible constraints (red lines) cluster across the DEP1–DEP2 linker region in full-length P-Rex1 indicating potential conformational flexibility across this interface. Dashed line (black) indicates position of an intrinsically disordered loop (IDL) and the FDR is indicated for each dataset.

**Extended Data Fig. 5:**
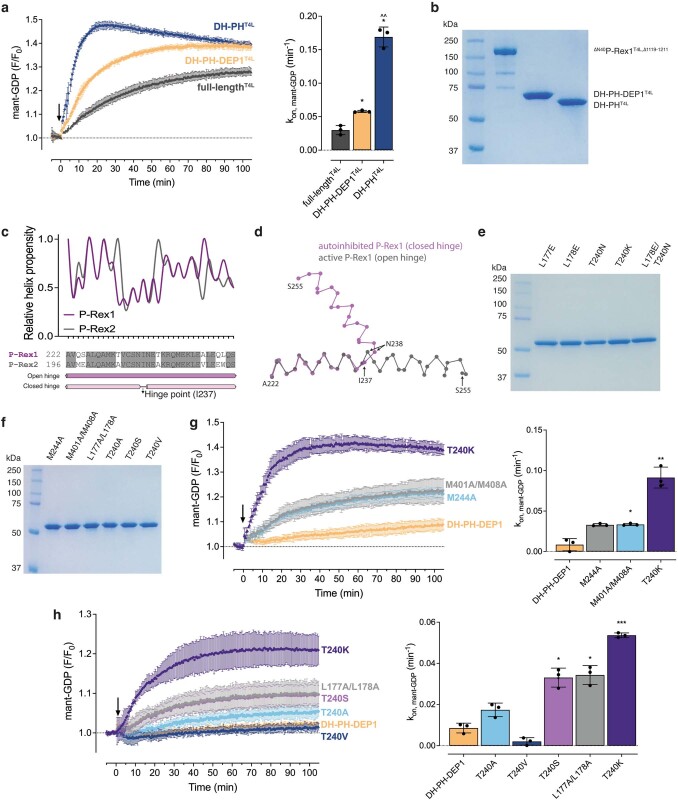
GEF activity of T4L P-Rex1 insertion variants or its domains and validation of DH-PH-DEP1 structure via mutagenesis. **a**. GEF activity of P-Rex1^T4L^ increases upon truncation of the C-terminal domains in a comparable pattern to wild-type P-Rex1. Activity of ^ΔN40^P-Rex1^T4L,Δ1119-1211^, DH-PH-DEP1^T4L^, and DH-PH^T4L^ variants monitored at 100 nM using mant-GDP activity assay. For timecourse graphs, symbols show mean and error bars show S.D. of n = 3 independent experiments conducted in duplicate. For bar graphs, symbols show rate constant (k_obs_) from independent experiments, bars show mean and error bars show S.D. (n = 3). * p < 0.05 versus full-length^T4L^ (p = 0.0392 for DH-PH-DEP1^T4L^ and p = 0.0141 for DH-PH^T4L^); ^^ p = 0.0097 versus DH-PH-DEP1^T4L^; repeated measures one-way ANOVA with Tukey’s multiple comparisons test. **b**. Coomassie-stained SDS-PAGE analysis of the indicated P-Rex1^T4L^ variants. **c**. Plot of relative α-helix propensity^49^ of each residue in the DH domain hinge helix for P-Rex1 and P-Rex2. A loss of α-helical propensity is observed in conserved residues surrounding the hinge-point (Ile237). **d**. Alignment of the DH domain hinge α_6_-helix in the autoinhibited (closed) structure and the active (open) conformation (PDB 4YON^13^). Structure in ball and stick format with the Cα atoms as spheres. **e-f**. Coomassie-stained SDS-PAGE analysis of the indicated P-Rex1 DH-PH-DEP1 mutants. **g-h**. GEF activity of the indicated DH-PH-DEP1 mutants. Activity of indicated P-Rex1 variants monitored at 20 nM using mant-GDP activity assay. For timecourse graphs, symbols show mean and error bars show S.D. of n = 3 independent experiments conducted in duplicate. For bar graphs, symbols show rate constant (k_obs_) from independent experiments, bars show mean and error bars show S.D. (n = 3). * p < 0.05, ** p < 0.01, and *** p < 0.001 versus DH-PH-DEP1 (p = 0.0431 for M401A/M408A, p = 0.0063 for T240K, p = 0.0398 for T240S, p = 0.0355 for L177A/L178A and p = 0.0009 for T240K), repeated measures one-way ANOVA with Dunnett’s multiple comparisons test. Numerical data for graphs in **a**, **g** and **h** are available as source data. Source data

Extensive crosslinking mass spectrometry (MS) of the DH-PH-DEP1 domains, in the absence or presence of T4L, revealed crosslinks in excellent agreement with the closed conformation observed in our structure (Extended Data Fig. 4 and Supplementary Data 1). Conversely, crosslinks frequently exceeded the allowable constraint distance (<30 Å between atoms^16^) when modeled on the active conformation (Extended Data Fig. 4 and Supplementary Data 1). Furthermore, P-Rex1^T4L^ variants maintained a comparable activation pattern upon DEP1 truncation as per the wild-type protein (Extended Data Fig. 5a,b).

Comparison of the closed DH-PH-DEP1 structure with the active DH-PH:Rac1 complex^13^ reveals a remarkable conformational change in the DH domain upon transition between the autoinhibited and active states (Fig. 1f–h). In the autoinhibited structure, the final DH domain α_6_-helix is broken into two halves at residue Ile237, forming a closed helix–turn–helix hinge (Fig. 1f–h and Extended Data Fig. 5c,d). The closed hinge enables the DEP1 domain to interact with the DH domain, forming a DEP1 latch that further stabilizes the closed conformation. Conformational transition to the active state requires a complete, roughly 126°, opening of the helix–turn–helix hinge to form a single extended α-helix (Fig. 1h). Hinge opening results in rotation of the DH domain away from the PH domain—this transition enables GTPase binding and activation. Primary sequence analysis of the hinge helix shows a conserved loss of α-helical propensity at the DH domain hinge site (Extended Data Fig. 5c).

Overall, the P-Rex1 DH domain helical hinge unraveling resembles calmodulin conformational switching^17^ and, to our knowledge, represents one of the most extensive conformational changes associated with G protein regulation observed so far. For example, the equivalent DH domain α_6_-helix of Trio bends around 30°, stabilizing an autoinhibitory DH-PH conformation sterically incompatible with RhoA binding^18^. Similarly, a bend in the ASEF DH domain α_6_-helix of around 45° supports an autoinhibited conformation in which an N-terminal SH3 domain sterically occludes Cdc42 binding^19^. Illustrating the mechanistic diversity of Rho-GEF family autoinhibition, the flexibility of the final DH domain α_6_-helix is not central to the autoinhibition of all Rho-family GEF proteins. For instance, Vav1 is autoinhibited by a short Ac domain helix at the N-terminus of the DH domain that sterically blocks the catalytic site^20^. The autoinhibited conformation of the Vav1 Ac helix is further stabilized by binding to an N-terminal CH domain^20^.

### A hinge-and-latch mechanism coordinates P-Rex1 autoinhibition

**Fig. 2:**
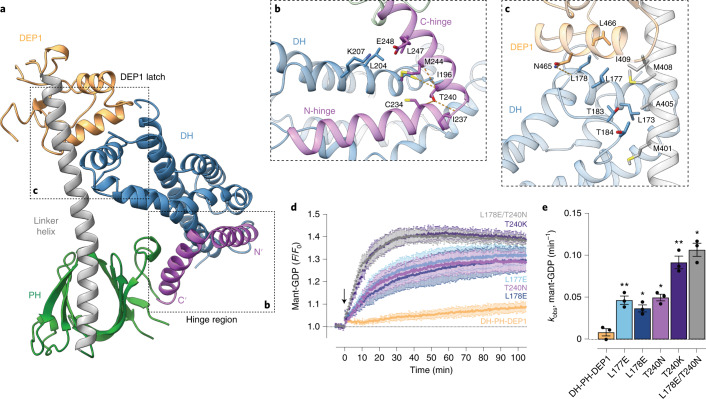
Structural basis of DH domain autoinhibition. **a**, Structure of DH-PH-DEP1 with the DH domain hinge region and the DEP1 latch region indicated. **b**, Close-up of the DH domain hinge region (rotated relative to **a**) highlighting the central Thr240 buried at the hinge point. Side chains of select interfacing residues are shown as stick and hydrogen bonds as dotted lines. **c**, Close-up of the DEP1 latch interface (rotated relative to **a**) highlighting the positioning of the DH domain in a cleft formed by the DEP1 domain and the PH-DEP1 linker helix. Side chains of select interfacing residues are shown as sticks and hydrogen bonds as dotted lines. **d**–**e**, GEF activity assay of the DH-PH-DEP1 domain demonstrating structure-guided hyperactivating DH-PH-DEP1 mutants. Activity of indicated P-Rex1 variants monitored at 20 nM using mant-GDP activity assay. Combination of DH hinge and DEP1 latch mutants provide an additive increase in P-Rex1 activity over either mutation alone. For timecourse graphs (**d**), symbols show mean and error bars show s.d. of *n* = 3 independent experiments conducted in duplicate. For bar graphs (**e**), symbols show rate constant (*k*_obs_) from independent experiments, bars show mean and error bars show s.d. (*n* = 3). *P < 0.05 and **P < 0.01 versus DH-PH-DEP1 (*P* = 0.0071 for L177E, *P* = 0.0115 for L178E, *P* = 0.0494 for T240N, *P* = 0.0079 for T240K, and *P* = 0.0219 for L178E/T240N), repeated measures one-way ANOVA with Dunnett’s multiple comparisons test. Numerical data for graphs in **d** and **e** are available as source data. Source data

Detailed structural analysis of the closed DH-PH-DEP1 structure reveals two main interfaces located at the DH hinge region and the DEP1 latch that support the autoinhibited conformation (Fig. 2a). At the DH domain hinge region, the conserved hinge α_6_-helix closes back on the main body of the DH domain, burying Thr240 in a cleft formed by DH domain helices α_3_ and α_5_ (Fig. 2b). Extending along the hinge helix, both Met244 and Leu247 are intercalated in the DH domain surface, further supporting the closed conformation (Fig. 2b). At the DEP1 latch, the largely hydrophobic α_4_-helix of the DH domain is positioned in a cleft formed by the PH-DEP1 linker helix and the DEP1 domain (Fig. 2c). The conserved α_4_-helix residues Leu177 and Leu178 bury a combined surface area of around 200 Å^2^. On the PH-DEP1 linker helix, the hydrophobic Met401 and Met408 further contribute to the interface by burying a combined total of around 160 Å^2^ (Fig. 2c).

To validate the structural basis of DH-PH-DEP1 autoinhibition, we initially tested several mutants designed to sterically hinder the closed conformation, but still support the active and open conformation. Substitution of Thr240 with the larger asparagine (T240N) or lysine (T240K) residues results in clear hyperactivation of P-Rex1 GEF activity (Fig. 2d,e and Extended Data Fig. 5e). At the DEP1 latch, replacement of either leucine residues L177 or L178 with glutamate also resulted in P-Rex1 hyperactivation (Fig. 2d,e and Extended Data Fig. 5e). This is probably due, at least in part, to disruption of the hydrophobic interface contributed by L177/L178 (on DH-α4) and M408/I409/L466 (on PH-DEP1 linker helix and DEP1) following mutation of the DH domain leucines to anionic residues. Moreover, we found that the combination of hinge and DEP1 latch mutations in the L178E/T240N double mutant provided an additive effect, increasing the activity of the P-Rex1 beyond either mutation alone (Fig. 2d,e and Extended Data Fig. 5e). In addition, mutation of several conserved hydrophobic interface residues (L177A/L178A, M244A, and M401A/M408A) to alanine largely resulted in DH-PH-DEP1 hyperactivation, highlighting their key role in the stabilization of the closed conformation (Extended Data Fig. 5f–h).

At the hinge region, the hydroxyl group of Thr240 hydrogen bonds to the main-chain of Cys234 or Ile237 to stabilize the closed conformation of the α_6_-helix (Fig. 2b). To analyze the contribution of side chain to backbone hydrogen bonding in stabilizing the α_6_-helix–turn-helix motif of the hinge region, we mutated Thr240 to alanine (T240A), valine (T240V) or serine (T240S). We found that both T240A or T240V were autoinhibited at a level comparable with that of wild-type DH-PH-DEP1 (Extended Data Fig. 5f,h). These data suggest that hinge-stabilizing T240 hydrogen bonding was not an overall requirement for P-Rex1 DH-PH-DEP1 autoinhibition. Further, T240S demonstrated notable hyperactivation, suggesting that the hydrophobic packing of the T240 methyl group contributes to the closed conformation. Interestingly, in P-Rex2 the equivalent residue to Thr240 is Ala214, indicating that the P-Rex2 α_6_-helix sequence is compatible with a similar autoinhibitory mechanism to that observed here for P-Rex1 (Extended Data Fig. 5c). Together, these data uncover a dual role for the DH hinge helix and the DEP1 latch regions in coordinating the large-scale rearrangement of the P-Rex1 DH-PH-DEP1 domains between the open and closed conformations.

### Cryo-EM structure of full-length P-Rex1

**Fig. 3:**
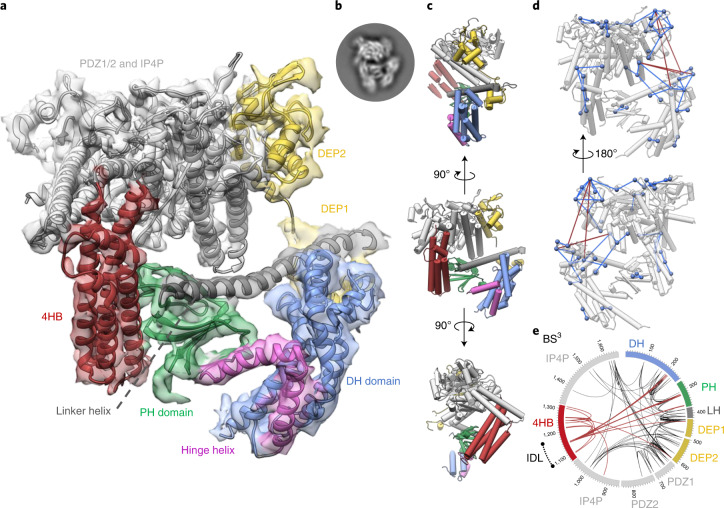
Cryo-EM structure of full-length autoinhibited P-Rex1. **a**, Cryo-EM map and structure of full-length P-Rex1. Map density for T4L is omitted for clarity (Extended Data Fig. 8) **b**, Inset, 2D classification from an equivalent molecular view for reference. **c**, Multiple indicated P-Rex1 views with domains highlighted. **d**, BS^3^ crosslink constraints (FDR = 6.8 %) from wild-type full-length P-Rex1 mapped onto the pipes-and-planks depiction of the full-length autoinhibited P-Rex1 model indicates numerous close range spatial constraints that are consistent with the modeled domain topology shown in **a**–**c**. Satisfied crosslinks are colored blue (<30 Å between lysine Cβ atoms), while crosslinks that exceed the maximum allowable distance (indicative of conformational flexibility) are in red (>30 Å between lysine Cβ atoms). Lysine Cβ atoms are shown as blue spheres. **e**, Circos plot of BS^3^ crosslinking mass spectrometry of full-length wild-type P-Rex1. Dashed line (black) indicates position of an IDL (located on the tip of the 4HB) removed in the cryo-EM construct.

**Extended Data Fig. 6:**
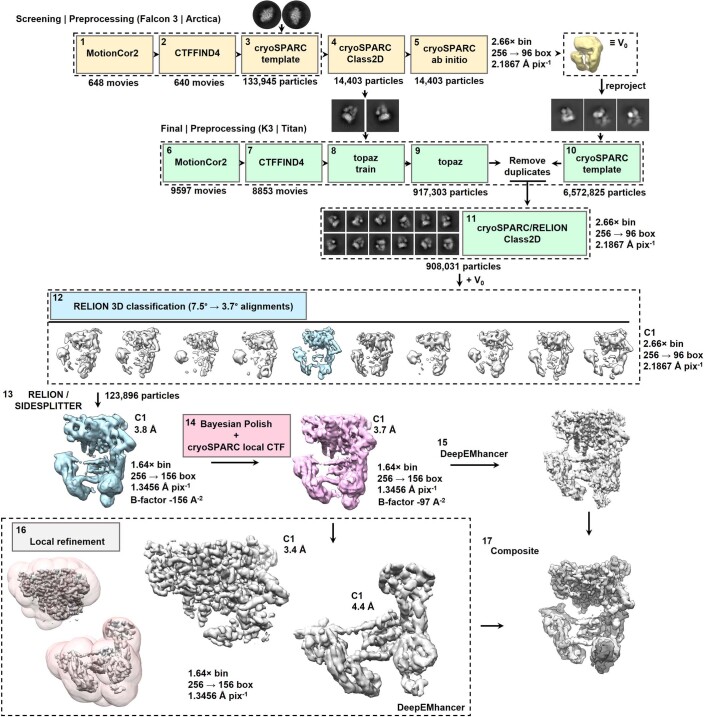
Cryo-EM image processing workflow and strategy. Initial data collection by Arctica and pre-processing indicated quality particles with density for the N-terminal module. An ab-initio volume and 2D class averages were used to bootstrap 3D classification and particle picking, respectively. A topaz^39^ model was trained using the top 100 micrographs ranked by number of quality particles. This was subsequently used to pick roughly 0.9 million particles. Template matching from re-projected templates of the ab-initio volume was performed yielding 6.5 million particles. Multiple rounds of 2D classification of this combined set yielded 0.9 million particles with good secondary structure. Two rounds of 3D classification with incrementally higher angular sampling was performed, and a single class was selected for refinement in RELION^34^ with SIDESPLITTER^40^. Local particle motion and CTF were further refined by Bayesian polishing and CTF refinement. Two modules of P-Rex1 showed notable flexibility and were therefore separated for local refinements. The final maps were sharped using DeepEMhancer^42^ (1.0) to suppress effects of anisotropy and visualise high-resolution features.

**Extended Data Fig. 7:**
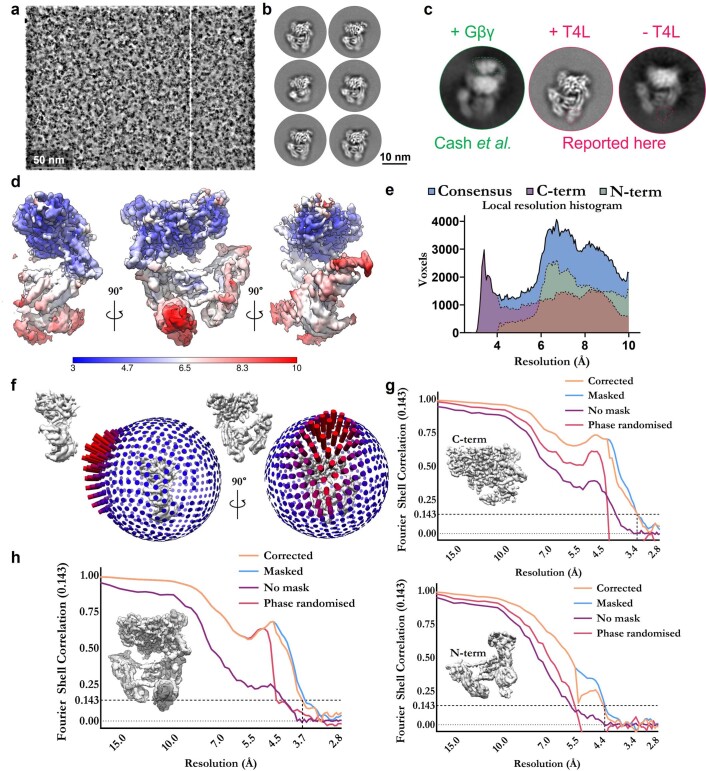
Cryo-EM reconstruction summary and validation. **a**. Warp^36^-denoised K3 micrograph of vitrified ^ΔN40^P-Rex1^T4L,Δ1119-1211^. Representative micrograph from a dataset of 9597 movies. **b**. RELION^34^ 2D class averages (no alignments) after particle polishing. **c**. Comparison of 2D class averages between autoinhibited P-Rex1 (without T4L, ^ΔN40^P-Rex1^Δ1119-1211, Δ990-1007^), P-Rex1 (with T4L, ^ΔN40^P-Rex1^T4L,Δ1119-1211^), and a single class from the previously published P-Rex1:Gβγ dataset (EMPIAR 10285)^15^ showing all three constructs form an equivalent closed domain conformation. **d**. Final composite reconstruction coloured according to local resolution as estimated by windowed FSC with 0.5 threshold criterion. **e**. Local resolution histogram showing distribution for both localised refinements and the consensus. **f**. Angular distribution of final reconstruction and corresponding views (left). **g**. Gold-standard Fourier Shell Correlation curves of independent half-maps (masked, unmasked, noise substitution corrected, and phase randomised) for both the C-terminal and N-terminal localised reconstructions. **h**. As in (g) for the full consensus reconstruction.

**Extended Data Fig. 8:**
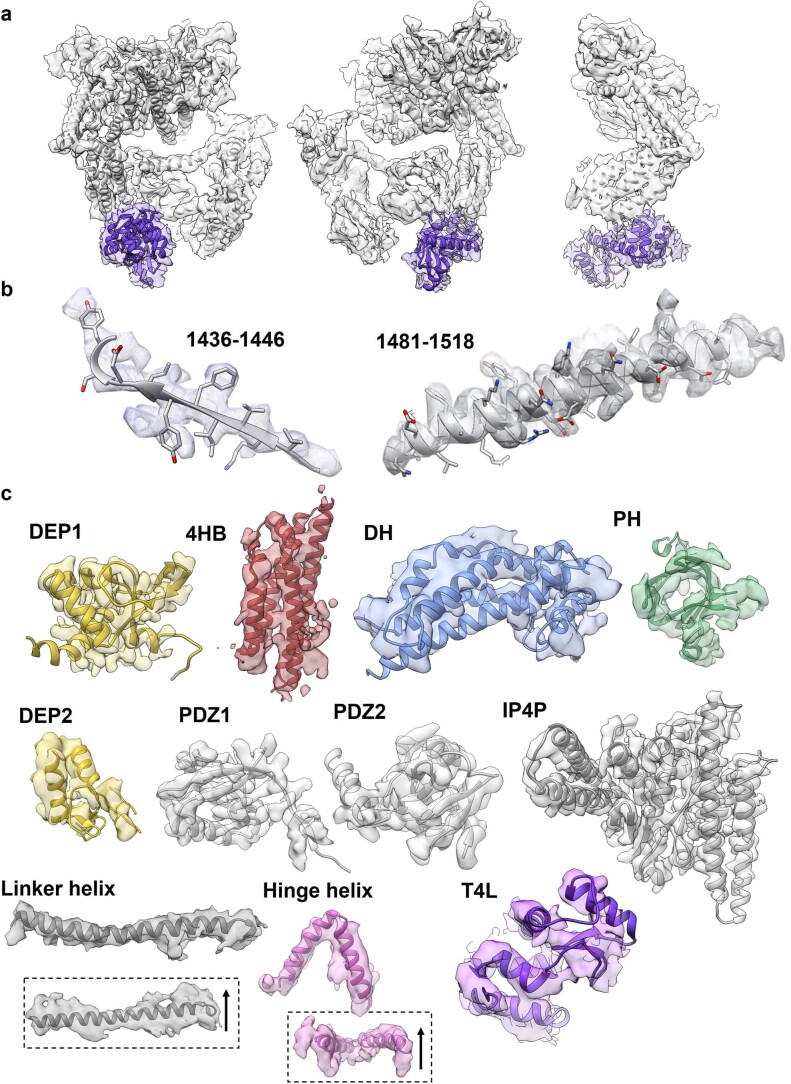
Density to model agreement. **a**. Three rotated views of the cryo-EM reconstruction of ^ΔN40^P-Rex1^T4L,Δ1119-1211^. Position of T4L is shown in purple. **b**. Select regions of high-resolution highlighting clear sidechain density **c**. Agreement between P-Rex1 model and cryo-EM density for each P-Rex1 domain. Regions of poor density due to anisotropy are shown with two views. Arrow indicates direction of the projection axis of the preferred orientation.

To investigate the mechanism by which Gβγ and PI(3,4,5)P_3_ cooperate synergistically to release P-Rex1 autoinhibition at the plasma membrane, we next purified full-length P-Rex1 and attempted to solve the entire autoinhibited structure by cryo-EM. However, the sample provided substantial challenges with a high degree of preferred orientation and a clear lack of features for the N-terminal domains in two-dimensional (2D) classes. We overcame these challenges by using a full-length P-Rex1^T4L^ construct incorporating deletions of the largely unstructured N-terminus (ΔN1–40) and 93 IP4P residues (Δ1119–1211) that are predicted to form an extended intrinsically disordered loop (IDL) region^21^. Despite ongoing limitations imposed by preferred orientation, we obtained a final P-Rex1 single-particle reconstruction at a nominal resolution estimated by Fourier shell correlation (FSC) to 3.8 Å (Fig. 3a–c, Table 2 and Extended Data Figs. 6 and 7). However, due to anisotropy effects, we estimate the true resolution to approximately 4.5 Å consistent with map features. Clear secondary structure features are present in the maps, allowing us to accurately dock our closed 3.2 Å DH-PH-DEP1 crystal structure and the 3.2 Å DEP2-PDZ1/2-IP4P^15^ structure into the reconstruction (T4L was omitted; Fig. 3a and Extended Data Fig. 8). An AlphaFold^21^ model of the previously unsolved four-helix bundle (4HB) IP4P region allowed us to build a complete model of autoinhibited P-Rex1. Crosslink MS constraints from full-length P-Rex1 without T4L or loop truncations confirmed the domain topology of the cryo-EM model (Fig. 3d,e, Extended Data Fig. 4 and Supplementary Data 1). Further, comparisons between P-Rex1 (with and without T4L) to a single class from the previously published P-Rex1:Gβγ dataset (EMPIAR 10285)^15^ shows all three constructs form an equivalent closed domain conformation indicating that T4L does not artifactually induce the observed domain packing (Extended Data Fig. 7c).

**Table 2:** Cryo-EM data collection, refinement and validation statistics

	P-Rex1 (EMD-25524) (PDB 7SYF)	P-Rex1 (N-term) (EMD-25525)	P-Rex1 (C-term) (EMD-25526)
**Data collection and processing**			
Magnification	×105,000	×105,000	×105,000
Voltage (kV)	300	300	300
Electron exposure (e^–^/Å^2^)	51.9	51.9	51.9
Defocus range (μm)	−0.5 to −2.2	−0.5 to −2.2	−0.5 to −2.2
Pixel size (Å)	0.823	0.823	0.823
Symmetry imposed	C1	C1	C1
Initial particle images (no.)	7,490,128	7,490,128	7,490,128
Final particle images (no.)	123,896	123,896	123,896
Map resolution (Å)			
0.143^b^/0.5^a^ FSC threshold	4.2^a^	4.4^b^	3.4^b^
Map resolution range (Å)			
0.5 FSC threshold	3.5 to ~9.0	4.0 to ~9.0	3.2 to ~6.5
Reconstruction type	Consensus	Localized	Localized
**Refinement**			
Initial model used (PDB code)	6PCV, 7RX9, AF-Q8TCU6-F1		
Model resolution (Å)			
0.143 FSC threshold	3.5	6.7	3.2
Map sharpening B factor (Å^2^)	−116	−101	−214
Model composition			
Non-hydrogen atoms	11,157		
Protein residues	1,397		
*B* factors (Å^2^)			
Protein	138.69		
Root mean squared deviations			
Bond lengths (Å)	0.013		
Bond angles (°)	1.868		
**Validation**			
MolProbity score	1.23		
Clash score	01.52		
Poor rotamers (%)	1.22		
Ramachandran plot			
Favored (%)	96.09		
Allowed (%)	3.91		
Disallowed (%)	0		

^a^We take the conservative threshold of 0.5 to account for FSC inflation due to anisotropy.

**Extended Data Fig. 9:**
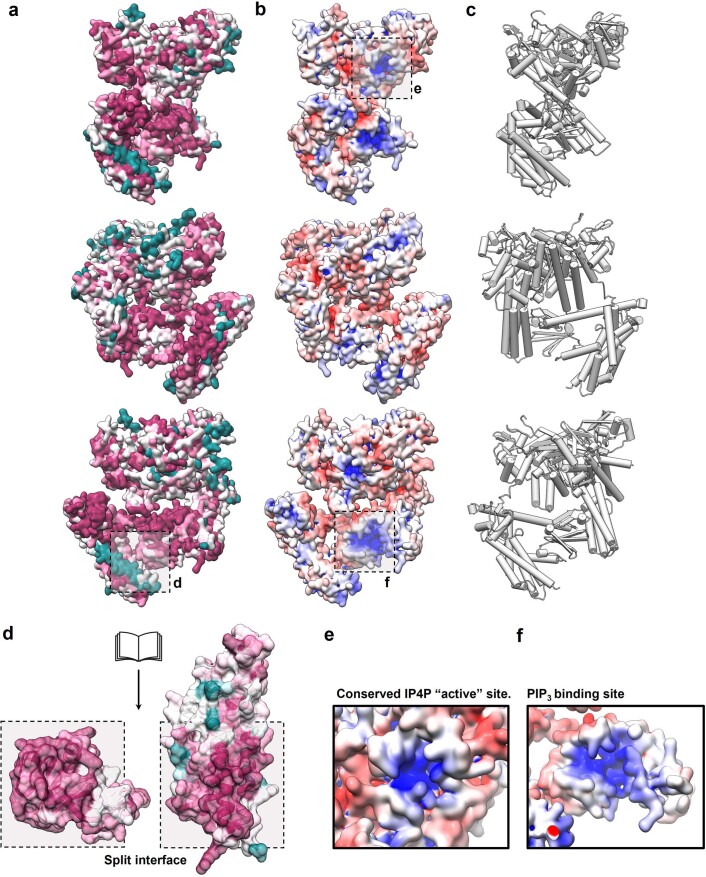
ConSurf and electrostatic surface analysis of full-length P-Rex1. **a**. Three 90° rotated views (surface rendering) of P-Rex1 coloured according to surface conservation^50^. **b**. Equivalent views coloured according to surface charge^51^. **c**. Pipes-and-planks depiction in equivalent orientations as in (a). **d**. Split interface of PH and 4HB domain interaction reveals a strongly conserved interface. **e**. Focused view of the IP4P conserved pseudo-active site. **f**. Focused view of the PH domain PI(3,4,5)P_3_ binding site. Highly conserved surface regions are observed on the DH, DEP1 and PH domains. These correlate with positively charged surface regions that may collectively mediate interactions with the negatively charged inner leaflet of the plasma membrane, as shown for the isolated P-Rex1 PH domain^14^.

Overall, full-length P-Rex1 is locked in a compact conformation with two distinct domain halves. The autoinhibited DH-PH-DEP1 domains form the N-terminal half that makes extensive contacts against the surface of the C-terminal DEP2-PDZ1/2-IP4P half (Fig. 3a–c). The interacting surfaces are conserved between species, with the PH domain buried in a cleft composed of the IP4P domain core and the prominent 4HB region (Fig. 3a and Extended Data Fig. 9). The DEP1–DEP2 linker seems to act as a flexible point to separate the N- and C-terminal P-Rex1 halves. Indeed, three-dimensional (3D) variability analysis (3DVA) of cryo-EM data demonstrates clear pivoting and bending motion across the two P-Rex1 halves about the 4HB-PH interface (Supplementary Video 1). Further, we frequently observed long-range crosslinks occurring between DEP1 and DEP2 indicative of conformational flexibility (Fig. 3d,e and Extended Data Fig. 4e,f). Together, these data suggest that DEP1 and DEP2 oscillate between a compact and an extended state (Fig. 3d,e and Supplementary Video 1).

### A two-layered mechanism of P-Rex1 autoinhibition

**Fig. 4:**
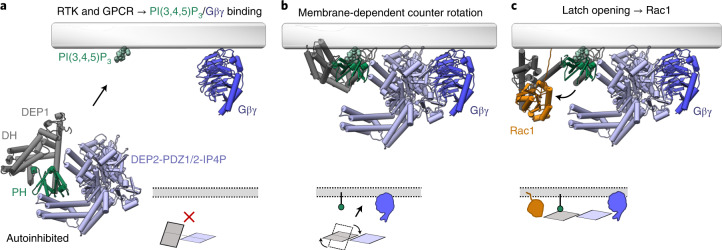
Model of synergistic P-Rex1 activation by Gβγ and PI(3,4,5)P_3_ at the plasma membrane. **a**, Cytoplasmic P-Rex1 is autoinhibited under basal conditions^3^. A hinge and latch mechanism locks the DH domain in a closed conformation where the PH domain sterically occludes the Rac1 binding site. In the autoinhibited P-Rex1 structure, the known membrane-binding regions are off-axis by around 90°. **b**, Concurrent binding of PI(3,4,5)P_3_ (ref. ^14^) and Gβγ^15^ at the membrane requires counter-rotation of the N-terminal (DH-PH-DEP1) and C-terminal (DEP2-PDZ1/2-IP4P) P-Rex1 halves to align the membrane-binding regions along a single plane. **c**, We hypothesize that the counter-rotation mechanism provides the conformational change required to release the DEP1 latch from the DH domain to activate P-Rex1. Structural analysis indicates that rotation-induced PH domain movement toward the membrane causes the PH-DEP1 linker helix to clash with the DH domain and likely triggers DEP1 latch release (Extended Data Fig. 10).

**Extended Data Fig. 10:**
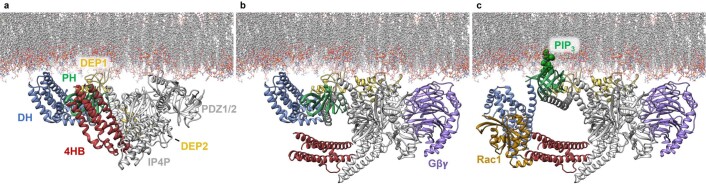
Proposed molecular mechanism of P-Rex1 synergistic activation by Gβγ and PI(3,4,5)P_3._ **a**. Autoinhibited P-Rex1 may transiently interact with the inner leaflet of the plasma membrane via DEP1, DEP2, PH or the IP4P positively charged surfaces. **b**. Collectively, binding of Gβγ and DEP1, DEP2 and IP4P requires the counter-rotation of the N-terminal module (DH-PH-DEP1) relative to the C-terminal module (DEP2-PDZ1/2-IP4P:Gβγ). Movement of the PH domain toward the membrane is essential to unlock the autoinhibited DH domain. **c**. Counter-rotation of N- and C-modules disengages the 4HB and PH interaction, freeing the PH domain to bind PI(3,4,5)P_3_. PI(3,4,5)P_3_ binding causes the DH domain latch to unlock, thereby releasing the DH domain to bind Rac1. Autoinhibited P-Rex1 is modelled by the full-length cryo-EM reconstruction and AlphaFold^21^ (4HB regions). The cryo-EM reconstruction of Gβγ:P-Rex1 DEP2-PDZ1/2-IP4P^15^ allows placement of Gβγ:DEP2-PDZ1/2-IP4P against the bilayer (revealing offset planes defined by N- and C-modules). The crystal structure of PH: PI(3,4,5)P_3_^14^, as well as functional data of DEP1/2^22^ membrane binding regions, guided the placement of the AlphaFold model of PH-DEP^21^ (kinked V-conformation) against the membrane. Lastly, superposition of the crystal structure of DH-PH:Rac1^13^ onto the membrane-anchored PH domain provides a model of the full active state. The Rac1 lipidation site provides additional short-range distance constraints on the placement of DH-PH:Rac1.

Notably, the results presented here allow us to postulate how cooperative binding of Gβγ and PI(3,4,5)P_3_ plasma membrane synergistically activates P-Rex1 (Fig. 4, Supplementary Video 2 and Extended Data Fig. 10). P-Rex1 has several membrane-binding regions located at the PH domain PI(3,4,5)P_3_-binding pocket^14^ and the lipid-binding β1-β2 loops of the DEP1 (ref. ^22^) and DEP2 (ref. ^15^) domains. In addition, structural alignment with the DEP2-IP4P:Gβγ^15^ complex provides the orientation of the Gβγ membrane-binding prenylation site. Intriguingly, the P-Rex1 PI(3,4,5)P_3_ and Gβγ membrane-binding prenylation sites are off-axis by around 90° in the autoinhibited structure. As such, we hypothesize that the cooperative binding of Gβγ and PI(3,4,5)P_3_ to P-Rex1 at the plasma membrane results in the counter-rotation of the N- and C-terminal P-Rex1 halves to align the membrane-binding regions along a single plane. This rotation would uncouple the IP4P and 4HB from the PH domain and likely trigger release of the catalytic DH domain to enable Rac1 binding. Gβγ and PI(3,4,5)P_3_ binding may act to allosterically relax the P-Rex1 autoinhibitory domain–domain interactions^15^ to promote counter-rotation and DEP1 latch release. Importantly, a counter-rotation mechanism rationalizes the requirement for membrane-bound prenylated Gβγ, rather than soluble Gβγ, to efficiently activate P-Rex1 (ref. ^3,15^). Although soluble Gβγ binds full-length P-Rex1, without the prenylated membrane anchor point, it would be unable to support the counter-rotation of the two P-Rex1 halves.

In summary, our integrative structural biology approach has allowed us to determine a full-length model of autoinhibited P-Rex1. We demonstrate a two-layered mechanism of P-Rex family autoinhibition. The activation of P-Rex1 requires synergistic binding of Gβγ and PI(3,4,5)P_3_ at the plasma membrane. We postulate that the first stage of activation requires a 90° counter-rotation of the two halves of P-Rex1. The second stage leads to the 126° opening of the DH hinge helix, releasing the steric inhibition of the PH domain. This conformationally open P-Rex1 can then catalyze GTPase activation. These two large-scale conformational changes within P-Rex1 enable a single protein fold to integrate signaling from two diverse receptor families.

## Methods

### Cloning

All enzymes used for cloning were purchased from New England Biolabs: Phusion High-Fidelity PCR Master Mix with HF Buffer (catalog no. M0531L), Antarctic Phosphatase (catalog no. M0289S), T4 DNA Ligase (catalog no. M0202L), *Bam*HI-HF (catalog no. R3136S), *Eco*RI-HF (catalog no. R3101S) and *XhoI* (catalog no. R0146S). RedSafe Nucleic Acid Staining Solution for staining DNA was provided by iNtRON Biotechnology (catalog no. 21141). DNA purification kits including Wizard Plus SV Minipreps DNA Purification System (catalog no. A1460) and Wizard SV Gel and PCR Clean-Up System (catalog no. A9282) were purchased from Promega.

DNA sequences encoding His-tagged P-Rex1 and its N-terminal domains DH-PH-DEP1 (residues 1–502) were cloned into pFastBac1 (Invitrogen) using the *Bam*HI/*Xho*I and *Bam*HI/*Eco*RI restriction sites, respectively. A TEV cleavage site was introduced between the His tag and P-Rex1 or DH-PH-DEP1. The T4 lysozyme (T4L: residues 2–161 with C54T and C97A mutations) insertion construct DH-PH-DEP1^T4L^ was generated by long PCR-based fusion^23^, resulting in the replacement by T4L of residues 306–322 in the loop connecting the β_3_ and β_4_ strands of the PH domain. This insertion construct served as a template for producing DH-PH^T4L^ and the N-terminally truncated construct ^ΔN40^DH-PH-DEP1^T4L^. Long PCR-based fusion of the coding sequences for ^ΔN40^DH-PH-DEP1^T4L^ and P-Rex1 C-terminal half (residues: 503–1659) with deletion of the longest loop in the IP4P domain (Δ1119–1211), which was identified by AlphaFold prediction^21^, yielded the construct ^ΔN40^P-Rex1^T4L,Δ1119–1211^ for structural determination of full-length P-Rex1 by cryo-EM. All constructs were verified by sequencing. We performed mutagenesis of DH-PH-DEP1 through gene synthesis (GenScript) (for variants: L177E, L178E, T240N, T240K, L178E/T240N, M244A and M401A/M408A) or long PCR-based fusion (for variants: T240A, T240V, T240S and L177A/L178A) using the following primers:

T240A_forward 5′… CAAACATCAATGAAGCCAAGAGGCAGATGGAGAAGCTGGAGG …3′

T240A_reverse 5′… CATCTGCCTCTTGGCTTCATTGATGTTTGAACAAACGGTTTTCATAGCTTG …3′

T240V_forward 5′… CAAACATCAATGAAGTGAAGAGGCAGATGGAGAAGCTGGAGG …3′

T240V_reverse 5′… CCATCTGCCTCTTCACTTCATTGATGTTTGAACAAACGGTTTTCATAGCTTG …3′

T240S_forward 5′… CAAACATCAATGAATCAAAGAGGCAGATGGAGAAGCTGGAGG …3′

T240S_reverse 5′… CTCCATCTGCCTCTTTGATTCATTGATGTTTGAACAAACGGTTTTCATAGCTTG …3′

L177A/L178A_forward 5′… GTATGGCAGCAGGGGGCCGAAAGACTACAGATATTC …3′

L177A/L178A_reverse 5′… CTTTCGGCCCCCTGCTGCCATACAGGACAGCAGGAAGGCTCG …3′

For insect cell expression of P-Rex1 (DH-PH), residues 1–404 of P-Rex1 were cloned into the polyhedron multiple cloning site of pFastBacDual (Invitrogen), and residues 1–177 of Rac1 were cloned into the p10 multiple cloning site of the same vector^13^. The DH-PH^T4L^ construct was generated in a similar fashion. For expression of Rac1 alone, Rac1 (residues 1–177) was cloned into pGEXTEV, a modified version of pGEX-4T-1 (GE Healthcare) where the thrombin site is replaced with a TEV cleavage site^13^.

### Protein expression and purification

His-tagged P-Rex1 and DH-PH-DEP1 or their variants were expressed in Sf9 cells for 2.5 days following infection of each 1 l culture by 3 ml of baculovirus, which was produced by following the manufacturer’s protocol (Bac-to-Bac, Invitrogen). Cells were harvested by centrifugation, washed in a buffer containing 20 mM Tris, pH 8.0, 500 mM NaCl, 10% glycerol and 5 mM β-mercaptoethanol and stored at −80 °C until use. Thawed cells were resuspended in lysis buffer (20 mM Tris, pH 8.0, 500 mM NaCl, 10% glycerol, 5 mM β-mercaptoethanol, 20 mM imidazole), followed by addition of phenylmethylsulfonyl fluoride at a final concentration of 2 mM and one cOmplete Protease Inhibitor Cocktail tablet (Roche) for each 40 ml of resuspended cells. Following this step, cells were lysed by sonication, the lysate was cleared by centrifugation at 32,816*g* for 30 min and the resulting supernatant was loaded onto Ni-NTA resin (Qiagen) pre-equilibrated with lysis buffer. After binding for 1 h with agitation, the resin was washed with lysis buffer and protein eluted with elution buffer (20 mM Tris, pH 8.0, 500 mM NaCl, 10% glycerol, 5 mM β-mercaptoethanol, 500 mM imidazole). Subsequently, the protein sample was supplemented with TEV protease and Lambda protein phosphatase and then dialyzed against dephosphorylation buffer (20 mM Tris, pH 8.0, 200 mM NaCl, 10% glycerol, 2 mM dithiothreitol (DTT), 5 mM MnCl_2_) for the removal of His tag and dephosphorylation. After the overnight cleavage and dephosphorylation, protein was clarified, concentrated and purified by ion-exchange chromatography with buffer A (20 mM Tris, pH 8.0, 50 mM NaCl, 5% glycerol, 2 mM DTT) and buffer B (20 mM Tris, pH 8.0, 1 M NaCl, 5% glycerol, 2 mM DTT) and size exclusion chromatography in SEC buffer (20 mM HEPES, pH 8.0, 100 mM NaCl, 2 mM DTT). P-Rex1 and its variants were purified on a Mono Q 5/50 GL column and a Superdex 200 Increase 10/300 GL column (Cytiva), whereas DH-PH-DEP1 and its variants were purified on a Mono S 5/50 GL column and a Superdex 75 Increase 10/300 GL column (Cytiva). Fractions were pooled, concentrated and either used freshly or flash frozen in liquid nitrogen for long-term storage at −80 °C. DH-PH, DH-PH^T4L^, DH-PH-DEP1^T4L^ and ^ΔN40^DH-PH-DEP1^T4L^ were purified following the purification protocol for DH-PH-DEP1. Note that, for cryo-EM sample preparation of ^ΔN40^P-Rex1^T4L,Δ1119-1211^, TEV cleavage of the His tag was skipped as the presence of the short tag did not seem to have a noticeable effect on grid quality. Rac1 (1–177) was expressed overnight in *Escherichia coli* BL21(DE3) cells at 18 °C following isopropylthiogalactoside induction^13^. Cells were resuspended in lysis buffer containing 20 mM Tris, pH 8.0, 500 mM NaCl, 2 mM DTT and 2 mM EDTA and lysed by sonication. The lysate was cleared by centrifugation at 32,816*g* for 30 min, and the resulting supernatant was loaded onto glutathione-sepharose 4B (Genscript) pre-equilibrated with lysis buffer. After binding for 1.5 h with agitation, the resin was washed with lysis buffer and incubated overnight at 4 °C with TEV protease to cleave the GST tag. After the overnight cleavage, the protein was clarified, concentrated and purified on a HiLoad Superdex 75 16/60 size exclusion column (Cytiva) equilibrated with SEC Buffer (20 mM HEPES, pH 8.0, 100 mM NaCl and 2 mM DTT)^13^.

### Crystallization and structure determination

P-Rex1 ^ΔN40^DH-PH-DEP1^T4L^ crystals were grown at 20 °C by hanging drop vapor diffusion in 50 mM 2-(N-morpholino)ethanesulfonic acid, pH 6.0, 1.8 M (NH_4_)_2_SO_4_ and 5 mM magnesium acetate. A 0.75 μl sample of P-Rex1 ^ΔN40^DH-PH-DEP1^T4L^ in SEC Buffer at 5 mg ml^–1^ was mixed with 1.0 μl of precipitant. Crystals were flash cooled in liquid nitrogen in cryoprotectant consisting of 50 mM 2-(N-morpholino)ethanesulfonic acid, pH 6.0, 2 M (NH_4_)_2_SO_4_, 5 mM magnesium acetate and 3 M proline. X-ray data were collected at the MX2 microfocus beamline^24^ of the Australian Synchrotron at a wavelength of 0.95373 Å and temperature of 100 K. Data were processed and scaled using XDS^25^ and programs within the CCP4 (ref. ^26^) suite. The high-resolution cut-off was determined by the criteria of *CC*_1/2_ > 0.3 (Table 1)^27^. The P-Rex1 ^ΔN40^DH-PH-DEP1^T4L^ structure was solved by molecular replacement using P-Rex1 DH-PH domains (PDB 4YON, ref. ^13^) as the search model in Phaser. Iterative cycles of refinement and rebuilding were carried out using PHENIX Refine^28^ with local rebuilding in COOT^29^. The structure had no Ramachandran outliers, with 96.70% of residues in favored regions and 3.30% in allowed regions and a final MolProbity^30^ score of 1.29 (100th percentile).

### GEF activity assay

GEF activity of P-Rex1 or its domains (including variants) were measured by following a modified N-methylanthraniloyl (mant)-GDP (Invitrogen) exchange protocol^13^. Specifically, 2 μM Rac1 was equilibrated with 2 μM mant-GDP in reaction buffer (20 mM Tris, pH 7.4, 50 mM NaCl, 5 mM MgCl_2_, 1 mM DTT, 5% glycerol) for 30 min at room temperature. For each experiment, we performed duplicates for each sample. Three independent experiments were carried out for statistical analysis. The time interval for each cycle was 32 s, and the gain for mant-GDP fluorescence signal was adjusted to 35% of the allowed maximal value. Ten cycles were run to establish the baseline before the addition of P-Rex1 proteins or its domains (including variants) to a final concentration of 20 nM or 100 nM. Following this, data collection was resumed and mant-GDP exchange was monitored for another 240 cycles. Data were analyzed by calculating the *F*/*F*_0_ (change in mant-GDP fluorescence relative to the average baseline fluorescence for each condition) and then expressed relative to the buffer control at each time point. The rate constant (*k*_obs_) was determined using a ‘Plateau followed by one phase association’ equation in GraphPad Prism v.9.2.0 with the initial value of *X* at zero (*X*_0_) constrained to 0 min. Rate constants were determined for each independent experiment. Data are expressed as the mean ± s.d. from three independent experiments conducted in duplicate.

### Crosslinking MS

We performed crosslinking MS as described previously^31^, by adding BS^3^ (Thermo Fisher) crosslinker at a 1:100 molar ratio to between 0.5 and 5 µM P-Rex1 and P-Rex1 variants in 20 mM HEPES pH 8.0, 100 mM NaCl and 2 mM DTT. Samples were incubated at room temperature for 20 min before the addition of 50 mM Tris-HCl pH 8.0 to quench the reaction. Samples were snap-frozen in liquid nitrogen. To process samples for mass spectrometry, samples were denatured at 65 °C with 10 mM DTT for 30 min. After addition of 40 mM chloroacetamide, samples were incubated for 20 min at room temperature in the dark. A 1:100 (w/w) ratio of trypsin was added to the samples, and the samples were incubated overnight at 37 °C. Trypsin digestion was stopped using 1 % (v/v) formic acid, before samples were cleaned using OMIX C18 pipette tips (Agilent Technologies). Samples were stored in 0.1 % (v/v) formic acid before MS.

Samples were analyzed by liquid chromatography with tandem MS (LC-MS/MS) using a Dionex Ultimate 3000 RSLCnano system coupled onto an QExactive HF Hybrid Quadrupole-Orbitrap mass spectrometer (Thermo Fisher). Separation of tryptic peptides used an Acclaim PepMap RSLC analytical column (75 µm × 50 cm, nanoViper, C18, 2 µm, 100 Å; Thermo Scientific) and an Acclaim PepMap 100 trap column (100 µm × 2 cm, nanoViper, C18, 5 µm, 100 Å; Thermo Scientific), by increasing concentrations of 80 % (v/v) acetonitrile/0.1% (v/v) formic acid at a flow of 250 nl min^−1^ for 90 min. The mass spectrometer was operated in data-dependent acquisition mode. The MS1 resolution was set at 120,000 over a scan range of 375–2,000 *m*/*z*. The AGC target was set at 3.0 × 10^6^ with a maximum injection time of 118 ms. The 12 most abundant precursor peaks were selected for MS2 acquisition using a resolution of 60,000 and an AGC target of 5.0 × 10^5^ with a maximum injection time of 118 ms. pLink2 (ref. ^32^) was used to identify BS^3^ crosslinked peptides. Each dataset is derived from at least two repeats, and crosslinked peptides were considered for further analysis if they had an E*-*value of less than 10^−4^. Visual representations of crosslinked peptides were generated in Circos^33^. The maximum compatible crosslinker length was considered to be 30 Å, as previously defined^16^. To calculate the false discovery rate (FDR), crosslinks were rendered in 3D by mapping constraints to the Cβ atomic coordinates of lysines in the different atomic models. The FDR for each model was defined as the sum of forbidden crosslinks multiplied by their frequency of observation, divided by the total sum of all observed crosslinks.

### Cryo-EM sample preparation and data collection

Quantifoil R1.2/1.3 300 mesh UltraAu grids were glow discharged using a Pelco easiGlow instrument at 15 mA for 90 s. Freshly purified protein (3.5 μl at 0.18 mg ml^−1^) was applied immediately to the discharged grid and vitrified in liquid ethane using a Vitrobot Mk IV (Thermo Fisher Scientific) after blotting by hand using Fisherman Grade 1 filter paper. Temperature was maintained at 4 °C with the relative humidity at 100%.

Preliminary data were collected on a Talos Arctica (Thermo Scientific) operating at 200 kV with a 50 μm C2 aperture. Micrographs were acquired as described previously^31^, using a bottom mounted Falcon 3 direct electron detector. The detector was used in counting mode at a nominal magnification of ×150,000, corresponding to a calibrated physical pixel size of 0.94 Å. The electron dose rate was set to 0.67 electrons pixel^−1^ s^−1^ with a total exposure time of 65.52 s, yielding a total dose of 49.99 electrons Å^−2^. Automated collection was carried out using EPU v.2.0 with beam-shift to collect nine images per stage movement. Defocus range was set between −0.5 and −2.2 μm.

Final data were collected on a Titan Krios G1 (Thermo Scientific) operating at 300 kV with a 50 μm C2 aperture. Micrographs were acquired using a Gatan K3 direct electron detector in counting mode at a nominal EF-TEM magnification of ×105,000, corresponding to a calibrated physical pixel size of 0.823 Å. A Gatan GIF Quantum energy filter was used with a slit width of 10 eV. The electron dose rate was set to 7.641 electrons pixel^−1^ s^−1^ with a total exposure time of 5 s, yielding a total dose of 51.9 electrons Å^−2^. Automated collection was carried out using EPU v.2.12.0.2771 with beam-shift to collect 21 images per stage movement. Defocus range was set between −0.5 and −2.2 μm.

### Cryo-EM data processing

We performed initial sample screening on a Talos Arctica (Thermo Scientific) to assess particle orientation, quality and the impact of ice-thickness on sample behavior. Small datasets of roughly 600 movies were collected for various constructs and buffer conditions, amounting to 18 datasets and 34,637 movies in total. These included extensive attempts to overcome preferred orientation involving detergent screens (CHS, LMNG, CTAB and DDM), 15–30° tilt data-collection schemes and multiple protein constructs. A combination of RELION^34^ (v.3.2), cryoSPARC^35^ (v.3.2.0) and WARP^36^ (v.1.0.9) was used to assess the data. An initial dataset of ^ΔN40^P-Rex1^T4L,Δ1119-1211^ yielded particles with clear density for the N-terminal regions. We therefore collected a large dataset of 9,597 movies and a further 3,092 movies at 30° tilt. The latter failed to produce classes with sensible features and further did not improve reconstructions of P-Rex1 when merged with nontilted data. All further processing excluded the tilted data.

All 9,597 movies were corrected for beam-induced motion and compensated for radiation damage within MotionCor2 (ref. ^37^) (v.1.1.0). Aligned dose-fractionated movie frames were summed for further processing. The contrast transfer function parameters were estimated with CTFFIND^38^ (v.4.1.8); 744 movies were discarded due to optical aberrations or poor quality. An ab initio volume was generated in cryoSPARC^35^ from 14,403 classified particles curated from the preliminary data. A topaz^39^ model was trained using 100 micrographs that possessed the greatest number of particle coordinates after initial cleaning and 2D classification. This model was subsequently used to pick 917,303 particles from the entire dataset. Secondly, reprojections of the ab initio volume were used for template matching in cryoSPARC^35^ yielding 6,572,825 particles. The union of these coordinates was taken after removal of duplicates.

We performed multiple rounds of 2D classification in RELION^34^ and cryoSPARC^35^ to remove denatured particles lacking signal for the N-terminal region and enrich particles with secondary structure features. Extracted particles were binned by extraction in a 256-pixel box down sampled to 96-pixels, corresponding to 2.1867 Å pixel^−1^. A cleaned set of 908,031 particles were used for 3D classification in RELION^34^. Initially, 7.5° angular sampling was performed for 15 iterations, followed by a further 15 at 3.75°. Classification with angular sampling was essential to suppress effects of the strong preferred orientation. Qualitatively, we found other classification schemes (such as classification without angular sampling) failed to optimize for isotropy, yielding classes that consisted only of particles of high quality but not uniformly sampled. A single class consisting of 123,896 particles yielded further reconstructions that were most isotropic, and thus these particles were selected for further processing. Particles were resampled to 1.3456 Å pixel^−1^.

Refinement in RELION^34^ with SIDESPLITTER^40^ (v.1.3) yielded a map of 3.8 Å nominal resolution with appropriate secondary structure features that had suppressed anisotropic artefacts. Bayesian polishing and local contrast transfer function (CTF) refinement were performed to correct for per-particle variation of motion and CTF parameters in RELION^34^ and cryoSPARC^35^, respectively. Refinement of higher order optical aberrations did not improve the maps. Any 3D continuous conformational heterogeneity was assessed by 3DVA in cryoSPARC^41^, which indicated the N-terminal and C-terminal modules of P-Rex1 were dynamic and flexible about the DEP1–DEP2 boundary. Therefore, localized reconstructions centered on each P-Rex1 half were performed in cryoSPARC^35^ local refinement with Gaussian priors to suppress divergence. These refinements yielded improved map quality and nominal resolutions of 3.4 Å and 4.4 Å for the C-terminal (DEP2-IP4P) and N-terminal (DH-DEP1) regions, respectively. Importantly, due to inflation of the FSC caused by anisotropic particle distribution, the true resolution is anticipated to be lower. We estimate the resolution to be roughly 4.5 Å on average consistent with secondary structure features and bulky side chains visible in the map.

Finally, comparisons between P-Rex1 constructs, including the previously published P-Rex1:Gβγ dataset^15^ (EMPIAR 10285), were performed as described above. Briefly, EMPIAR 10285 data were processed according to standard cryo-EM single-particle analysis. Preprocessed particles were imported to cryoSPARC and subject to multiple rounds of 2D classification. Full-length autoinhibited P-Rex1:Gβγ classes were readily apparent, albeit comparatively rare consistent with our observations on P-Rex1 alone (without T4L).

All maps were sharpened to visualize high-resolution features and assess map quality using DeepEMhancer^42^. DeepEMhancer was found to suppress effects of anisotropy and consistently revealed secondary structure features, such as individual β-strands, where traditional sharpening methods failed (yielding over sharpened noisy maps). Conversions between software were performed with EMAN^43^ (v.2.2), with code written inhouse or by pyem. Local resolution was estimated by the windowed blocres FSC method as implemented in cryoSPARC^35^ with a 0.5 threshold.

### Model building

An initial model of autoinhibited full-length P-Rex1 was generated by rigid body docking the cryo-EM structure of Gβγ:DEP2-PDZ1/2-IP4P^15^ (PDB 6PCV) and the DH-PH-DEP1^T4L^ (PDB 7RX9) crystal structure directly into the cryo-EM reconstruction. The P-Rex1 AlphaFold^21^ model (AF-Q8TCU6-F1) was used to build the previously unresolved 4HB. These models were combined into a single chain, and unresolved loops were deleted. All atom constraints and secondary structure constrains were applied in ISOLDE^44^, and the model was flexibly refined into the cryo-EM density. This limited the movement of side chains from the predefined coordinates of the higher resolution structures but enabled improved fit of the AlphaFold^21^ model and resolved intradomain clashes. Ultimately, T4L was not modeled owing to extensive conformational dynamics in this region of the reconstruction (overall the poorest quality density) and since T4L was not resolved in the DH-PH-DEP1^T4L^ (PDB 7RX9) crystal structure.

A model of membrane-bound Gβγ:PI(3,4,5)P_3_:P-Rex1 was generated by placing P-Rex1 DEP2-IP4P:Gβγ^15^ against the bilayer according to the lipid/Gβγ interface defined by Gβγ:GPCR structures (6N4B, 6QNO). Further, the DEP2 membrane-binding loop and the IP4P charged interface were used as constraints to define the plane of the plasma membrane. This docking revealed counter-rotation of the DH-PH-DEP1 domains was necessary to define a single membrane-bound interface. Notably, the AlphaFold^21^ model of PH-DEP1 predicts a kinked, V-shape conformation whereby the PH and DEP1 domains define a single plane. In this state, the PH domain is raised relative to the autoinhibited conformation, which we interpreted as a lower energy state that was consistent with a PI(3,4,5)P_3_ bound conformation. This model was docked against the bilayer according to the inositol-(1,3,4,5)-tetrakisphosphate:PH^14^ crystal structure (PDB 5D3Y) and the DEP1 membrane-binding loop. Thus, the DEP1, DEP2, PH, IP4P and Gβγ membrane-binding interfaces were satisfied.

Next, the fully open active state of P-Rex1 was modeled by superimposing the Rac1:DH-PH^13^ crystal structure (PDB 4YON) onto the PH domain of the PI(3,4,5)P_3_ bound model. Lastly, the lipidation site of Rac1 provides a final constraint on the placement of models relative to the membrane, in agreement with the full model. Videos were made in USCF Chimera (v.1.14)^45^.

### Reporting summary

Further information on research design is available in the Nature Research Reporting Summary linked to this article.

## Online content

Any methods, additional references, Nature Research reporting summaries, source data, extended data, supplementary information, acknowledgements, peer review information; details of author contributions and competing interests; and statements of data and code availability are available at 10.1038/s41594-022-00804-9.

## Supplementary information

Reporting Summary

Supplementary Video 1 Continuous conformational (3DVA) heterogeneity analysis. A single mode of continuous linear conformational embedding solved by 3DVA analysis in cryoSPARC. Bending motion is observed about the 4HB/PH interface, causing dynamic opening and closing of the DEP1/DEP2 domains. In solution (due to autoinhibition), we do not anticipate to observe the full range of possible motion between the N- and C-modules. However, 3DVA analysis provides support for uncoupling of the N/C-domain halves upon activation by PI(3,4,5)P_3_ and Gβγ.

Supplementary Video 2 Proposed molecular mechanism of P-Rex1 synergistic activation by Gβγ and PI(3,4,5)P_3_ (animated). See legend to Extended Data Fig. 10.

Supplementary Data 1 Crosslinking mass spectrometry of P-Rex1. The distance between lysine Cβ atoms are noted.

## Data Availability

Coordinates were deposited with the Protein Data Bank (https://www.rcsb.org) with accession numbers PDB 7RX9 (DH-PH-DEP1 crystal structure) and PDB 7SYF (P-Rex1 cryo-EM structure). The 3D cryo-EM density map was deposited with the Electron Microscopy Data Bank (https://www.ebi.ac.uk/pdbe/emdb/) under accession numbers EMD-25524, EMD-25525 and EMD-25526. Coordinates used in analysis 4YON, 7SYF, 6N4B, 6QNO, 5D3Y and 6PCV are available at the Protein Data Bank (https://www.rcsb.org). The mass spectrometry proteomics data have been deposited with the ProteomeXchange Consortium via the PRIDE^46^ partner repository with the dataset identifier PXD034327. Source data are provided with this paper.

## Source data

Source Data Fig. 1 Numerical source data.
Source Data Fig. 2 Numerical source data.
Source Data Extended Data Fig. 1 Numerical source data.
Source Data Extended Data Fig. 5 Numerical source data.
