# Supplementary material for: Structure of the metastatic factor P-Rex1 reveals a two-layered autoinhibitory mechanism
Source: Nat Struct Mol Biol. 2022 Jul 21;29(8):767–73. doi: 10.1038/s41594-022-00804-9 (PMC9371973; doi:10.1038/s41594-022-00804-9)
Supplement: Supplementary file 1 — Reporting Summary [file 41594_2022_804_MOESM1_ESM.pdf]

nature portfolio

Corresponding author(s): Andrew M Ellisdon, Yong-Gang Chang

Last updated by author(s): Jun 6, 2022

Reporting Summary

Nature Portfolio wishes to improve the reproducibility of the work that we publish. This form provides structure for consistency and transparency in reporting. For further information on Nature Portfolio policies, see our Editorial Policies and the Editorial Policy Checklist.

Statistics

For all statistical analyses, confirm that the following items are present in the figure legend, table legend, main text, or Methods section.

n/a	Confirmed	
☐	☒	The exact sample size (*n*) for each experimental group/condition, given as a discrete number and unit of measurement
☒	☐	A statement on whether measurements were taken from distinct samples or whether the same sample was measured repeatedly
☐	☒	The statistical test(s) used AND whether they are one- or two-sided<br>*Only common tests should be described solely by name; describe more complex techniques in the Methods section.*
☒	☐	A description of all covariates tested
☒	☐	A description of any assumptions or corrections, such as tests of normality and adjustment for multiple comparisons
☐	☒	A full description of the statistical parameters including central tendency (e.g. means) or other basic estimates (e.g. regression coefficient) AND variation (e.g. standard deviation) or associated estimates of uncertainty (e.g. confidence intervals)
☐	☒	For null hypothesis testing, the test statistic (e.g. *F*, *t*, *r*) with confidence intervals, effect sizes, degrees of freedom and *P* value noted<br>*Give P values as exact values whenever suitable.*
☒	☐	For Bayesian analysis, information on the choice of priors and Markov chain Monte Carlo settings
☒	☐	For hierarchical and complex designs, identification of the appropriate level for tests and full reporting of outcomes
☒	☐	Estimates of effect sizes (e.g. Cohen's *d*, Pearson's *r*), indicating how they were calculated

Our web collection on statistics for biologists contains articles on many of the points above.

Software and code

Policy information about availability of computer code

Data collection	EPU v2
Data analysis	Warp v1.0.9, cryoSPARC v3.2.0, MotionCor2 v1.1.0, CTFFIND v4.1.8, RELION v3.2, SIDESPLITTER v1.3, EMAN v2.2, Coot v0.9.5, Isolde v1.2, UCSF Chimera v1.14, Phenix v1.19.2, pLink v2, Circos v0.69, AlphaFold v2, GraphPad Prism v9.2.0, ccp4-7.1, XDS(BUILT2021), DeepEMhancer 1.0

For manuscripts utilizing custom algorithms or software that are central to the research but not yet described in published literature, software must be made available to editors and reviewers. We strongly encourage code deposition in a community repository (e.g. GitHub). See the Nature Portfolio guidelines for submitting code & software for further information.

Data

Policy information about availability of data

All manuscripts must include a data availability statement. This statement should provide the following information, where applicable:

- Accession codes, unique identifiers, or web links for publicly available datasets
- A description of any restrictions on data availability
- For clinical datasets or third party data, please ensure that the statement adheres to our policy

Coordinates were deposited in the Protein Data Bank (https://www.rcsb.org) with accession numbers PDB 7RX9 (DH-PH-DEP1 crystal structure), and PDB 7SYF (P-Rex1 cryo-EM structure). The three-dimensional cryo-EM density map was deposited into the Electron Microscopy Data Bank (https://www.ebi.ac.uk/pdbe/emdb/) under accession numbers EMDB 25524, EMDB 25525, EMDB 25526. Coordinates used in analysis 4YON, 7SYF, 6N4B, 6QNO, 5D3Y, 6PCV are available at the Protein Data Bank (https://www.rcsb.org). The mass spectrometry proteomics data have been deposited to the ProteomeXchange Consortium via the PRIDE partner repository with the dataset identifier PXD034327. Source data are provided with this paper.

March 2021

Field-specific reporting

Please select the one below that is the best fit for your research. If you are not sure, read the appropriate sections before making your selection.

☒ Life sciences ☐ Behavioural & social sciences ☐ Ecological, evolutionary & environmental sciences

For a reference copy of the document with all sections, see nature.com/documents/nr-reporting-summary-flat.pdf

Life sciences study design

All studies must disclose on these points even when the disclosure is negative.

Sample size	For cryo-EM studies, the number of movies (sample size) was determined by the number of final particles required to reach the stated resolution limit. One crystal was required for X-ray crystallography studies to obtain a complete data-set.
Data exclusions	No data excluded.
Replication	All attempts at replication (n=2-3 biological replicates) are included in the study, as stated in figure legends
Randomization	not applicable- no experimental groups were involved.
Blinding	not applicable- no group allocation was involved.

Reporting for specific materials, systems and methods

We require information from authors about some types of materials, experimental systems and methods used in many studies. Here, indicate whether each material, system or method listed is relevant to your study. If you are not sure if a list item applies to your research, read the appropriate section before selecting a response.

Materials & experimental systems

n/a	Involved in the study	
☒	☐	Antibodies
☐	☒	Eukaryotic cell lines
☒	☐	Palaeontology and archaeology
☒	☐	Animals and other organisms
☒	☐	Human research participants
☒	☐	Clinical data
☒	☐	Dual use research of concern

Methods

n/a	Involved in the study	
☒	☐	ChIP-seq
☒	☐	Flow cytometry
☒	☐	MRI-based neuroimaging

Eukaryotic cell lines

Policy information about cell lines

Cell line source(s)	Spodoptera frugiperda 9 cells (Thermo Fisher, 11496-015)
Authentication	Not authenticated
Mycoplasma contamination	Not tested
Commonly misidentified lines (See ICLAC register)	No commonly misidentified cell lines were used in this study
